# Simultaneous determination of diquat and its two primary metabolites in rat plasma by ultraperformance liquid chromatography–tandem mass spectrometry and its application to the toxicokinetic study

**DOI:** 10.1007/s11419-022-00623-z

**Published:** 2022-04-12

**Authors:** Zhengsheng Mao, Youjia Yu, Hao Sun, Chao Wu, Qiaoyan Jiang, Chunyan Chu, Chongwen Zhao, Yujie Zhou, Jinsong Zhang, Yue Cao, Feng Chen

**Affiliations:** 1 Department of Forensic Medicine, Nanjing Medical University, Nanjing, Jiangsu 211166 People’s Republic of China; 2 Department of Emergency, Jiangsu Province Hospital, The First Affiliated Hospital of Nanjing Medical University, Nanjing, China; 3 Department of Emergency, Suqian Hospital of Nanjing Drum Tower Hospital Group, The Affliated Suqian Hospital of Xuzhou Medical University, Suqian, China; 4 Key Laboratory of Targeted Intervention of Cardiovascular Disease, Collaborative Innovation Center for Cardiovascular Disease Translational Medicine, Nanjing Medical University, Nanjing, Jiangsu 211166 People’s Republic of China

**Keywords:** Diquat, Diquat dipyridone, Diquat monopyridone, HILIC column, UPLC–MS/MS

## Abstract

**Purpose:**

This study aimed to develop and validate an ultraperformance liquid chromatography–tandem mass spectrometry to simultaneously determine diquat (DQ) and its two primary metabolites in rat plasma and its application to the toxicokinetic study.

**Method:**

The chromatographic separation of DQ and its two primary metabolites was performed with hydrophilic interaction chromatography column by adding formic acid and ammonium acetate in mobile phase in stepwise elution mode. DQ and its two primary metabolites were detected by liquid chromatography–tandem mass spectrometry in positive mode.

**Results:**

The lower limit of quantification ranging from 0.3 to 3.0 ng/mL for DQ and its two primary metabolites was achieved by using only 50 μL of rat plasma. The maximum concentration (*C*_max_) was 977 ng/mL, half-life (*t*_1/2_) was 13.1 h, and area under the plasma concentration–time curve (AUC_0–t_) was 2770 h*ng/mL for DQ, *C*_max_ was 47.1 ng/mL, *t*_1/2_ was 25.1 h, and AUC_0–t_ was 180 h·ng/mL for diquat monopyridone (DQ-M) and *C*_max_ was 246 ng/mL, *t*_1/2_ was 8.2 h, and AUC_0–t_ was 2430 h·ng/mL for diquat dipyridone (DQ-D), respectively.

**Conclusions:**

The validated method was shown to be suitable for simultaneous determination of diquat and its two primary metabolites in rat plasma. This study is the first to study the toxicokinetics of DQ and its two primary metabolites.

**Supplementary Information:**

The online version contains supplementary material available at 10.1007/s11419-022-00623-z.

## Introduction

Diquat (DQ) is a non-selective contact herbicidal active ingredient used as a general herbicide to control weeds. At least 60 cases of DQ poisoning worldwide were reported in scientific literature [1]. DQ commonly targets the kidney, causing tubular necrosis in patients with DQ poisoning. Brain involvement of DQ poisoning has been reported recently in a lethal DQ poisoning case [2]. The toxicokinetics of DQ, especially its main metabolites, are still unclear.

Fuke et al. [3] reported that DQ could be metabolized to DQ monopyridone (DQ-M) and DQ dipyridone (DQ-D) in rats and poisoned patients. There have been a few developed methods for determination of DQ in non-biological samples [4–6] or biological samples [7–9]. Oulkar et al. [5] developed a liquid chromatography-tandem mass spectrometry (LC–MS/MS) method for the determination of the residues of DQ in various fruit matrices. Tsao et al. [9] published a method for simultaneous determination of DQ and other three herbicides in postmortem blood and urine by LC–MS/MS. However, there was no method that could simultaneously determine DQ and its two primary metabolites in plasma by LC–MS/MS.

In this study, we aimed to develop a fast ultraperformance liquid chromatography (UPLC) –MS/MS method for the determination of DQ and its two primary metabolites in rat plasma. Then, this study was applied to the toxicokinetic study in rats after intragastric administration of DQ. To the best of our knowledge, this is the first trial to study the toxicokinetics of DQ and its two primary metabolites.

## Materials and methods

### Chemicals and reagents

**Fig.1:**
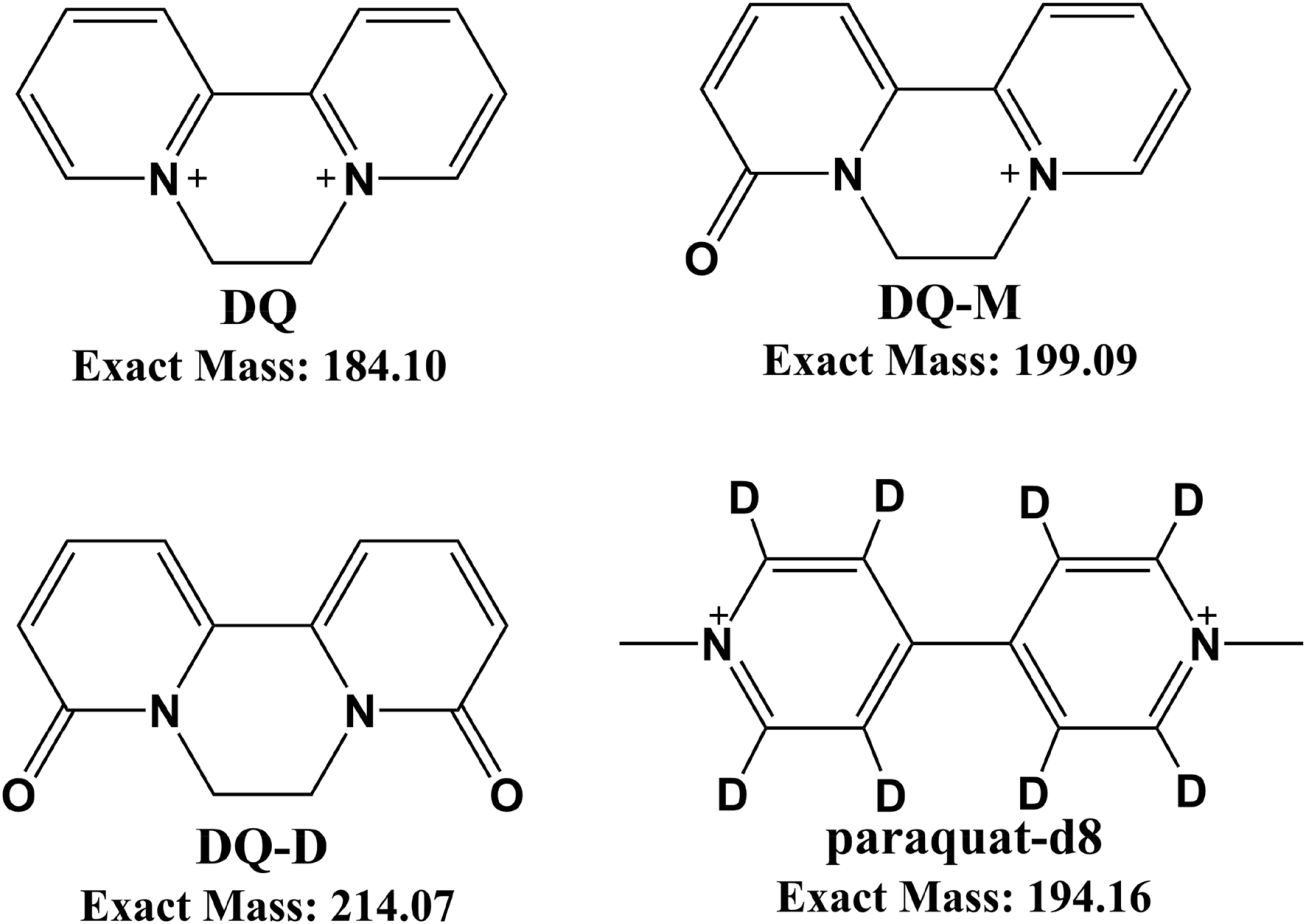
Structures of DQ, DQ-M, DQ-D and paraquat-d8 (internal standard: IS)

DQ, DQ-D, DQ-M and paraquat-d8 (internal standard: IS) were obtained from J&K Scientific Ltd. (Shanghai, China). The chemical structures of DQ, DQ-D, DQ-M and IS are shown in Fig. 1. The purities of all standards were above 95.0% (HPLC). Acetonitrile, methanol, formic acid and ammonium acetate of LC–MS grade were purchased from Sigma-Aldrich (St. Louis, MO, USA). Ultrapure water was prepared in our laboratory by ELGA LabWater system (ELGA Veolia, Bucks High Wycombe, UK).

### UPLC–MS/MS instrumentation and condition

UPLC–MS/MS analyses were performed with a TQ-S Micro (Waters Corp., Milford, MA, USA) triple quadrupole mass spectrometer in the electrospray ionization (ESI) mode. Chromatographic separation was carried out using a CORTECS^®^ UPLC^®^ HILIC (100 mm × 2.1 mm, 1.6 μm) column (Waters Corp., Milford, MA, USA) at 40 °C. The mobile phase composed of a mixture of 10 mM ammonium acetate with 0.5% formic acid (water phase) and acetonitrile (organic phase) was used at a flow rate of 0.35 mL/min. The stepwise elution was as follows: 70% organic phase (0–0.50 min), 30% organic phase (0.51–1.60 min), 70% organic phase (1.61–2.50 min). The source gas (nitrogen) flow was 660 L/h, cone flow was 33 L/h, desolvation temperature was at 460 °C, cone voltage was 35 V, and capillary voltage was 1.0 kV. The optimized mass parameters for the determination of DQ, DQ-D, DQ-M and IS in positive ion mode are shown in Table 1. Data acquisition was controlled using the MassLynx software (Waters).

**Table 1:** Multiple reactions monitoring parameters for DQ, DQ-M, DQ-D and internal standard (IS)

Compound	Precursor ion (*m/z*)	Product ion (*m/z*)	Dwell time (ms)	Cone (V)	Collision energy (v)
DQ	183.1	156.6 ^a^	0.25	20	42
		129.5 ^b^	0.25	20	35
DQ-M	199.1	155.1 ^a^	0.25	20	33
		78.3 ^b^	0.25	20	45
DQ-D	215.0	171.3 ^a^	0.25	30	35
		153.3 ^b^	0.25	30	30
IS	194.1	179.2 ^a^	0.25	25	31
		82.1 ^b^	0.25	25	44

^a^Quantifies

^b^Qualifies

### Preparation of the standards and quality control samples

The standard stock solutions of DQ, DQ-D, DQ-M and IS at 1.0 mg/mL were prepared in a methanol/water (50:50, *v*/*v*) solution. The working solutions of DQ, DQ-D, DQ-M and IS for calibration and controls were subsequently prepared by appropriate dilution in a methanol/water (50:50, *v*/*v*) solution. The calibration standards were made at 3.0, 9.0, 30.0, 90.0, 300, 900 and 3000 ng/mL for DQ, 0.9, 3.0, 9.0, 30.0, 90.0, 300 and 900 ng/mL for DQ-D, 0.3, 0.9, 3.0, 9.0, 30, 90 and 300 ng/mL for DQ-M, respectively. IS working solution was prepared at 500 ng/mL. Quality control samples were prepared at 3.0, 9.0, 300 and 2700 ng/mL for DQ, 0.9, 3.0, 300.0 and 810 ng/mL for DQ-D, 0.3, 0.9, 90 and 270 ng/mL for DQ-M, respectively.

### Sample preparation

All frozen rat plasma samples were thawed at room temperature and vortex mixed. An aliquot of 50 μL of plasma was pipetted into a 1.5 mL Eppendorf tube. Then, 50 μL of IS working solution was added and vortex mixed. Then, 150 μL acetonitrile was added, vortex mixed and centrifuged at 2000 × g for 5 min. Then, 20 μL clear upper layer was diluted with 80 μL ultrapure water. Finally, 10 μL of clear upper layer was injected into the UPLC–MS/MS system.

### Method validation

The method validation was performed according to European Medicines Agency (EMA) guidelines and US Food and Drug Administration (FDA) guidelines for the validation of bioanalytical methods [10, 11].

### Selectivity and carryover

The selectivity was evaluated by analyzing blank rat plasma samples from six different sources and checking for endogenous substances with the DQ, DQ-D, DQ-M and IS. A selective method was accepted as a response of interfering peaks no more than 20% of the lower limit of quantification (LLOQ) for DQ, DQ-D, DQ-M and 5% for the IS. Carryover was tested by three consecutive injections of a blank rat plasma sample after the injection of a high concentration calibrator. Carryover in the blank rat plasma sample following the highest concentration calibrator was not greater than 20% of the LLOQs of DQ, DQ-D, DQ-M, respectively.

## Linearity and lower limit of quantification

Calibration standards with DQ/IS, DQ-D/IS and DQ-M/IS at seven concentrations in blank rat plasma were extracted and analyzed by UPLC–MS/MS. Each calibration curve plotted by the area ratio (*y*) of analyte/IS versus the nominal concentration (*x*) was analyzed individually with a weighting factor of 1/*x*^2^. The linearity was evaluated over a range of 3.0–3000 ng/mL for DQ, 0.9–900 ng/mL for DQ-D and 0.3–300 ng/mL for DQ-M, respectively. A correlation coefficient (*r*) value > 0.99 was desirable. The LLOQs for DQ, DQ-D and DQ-M in rat plasma samples were defined as the lowest concentration at least ten times the response compared to the blank response.

### Accuracy and precision

The precision and accuracy were evaluated at four concentration levels (LLOQ, low quality control, middle quality control and high quality control) by analyzing six different replicates over three different days (*n* = 18 replicates). The accuracy and relative standard deviation (RSD) at each concentration level were expected to be within ± 15% except for the LLOQ where it could be ± 20%.

## Extraction recovery and matrix effect

Extraction recovery was determined by comparing the peak areas of DQ, DQ-D, DQ-M or IS obtained from rat plasma samples spiked before extraction to those spiked after extraction. The matrix effects were determined by calculating the ratio of the peak area in the presence of matrix (measured by analyzing the blank matrix spiked with DQ, DQ-D, DQ-M or IS) to the peak area in the absence of matrix (pure solution of the DQ, DQ-D, DQ-M or IS: neat sample).

### Stability

Stock solutions of DQ, DQ-D, DQ-M and IS were checked for stability (14 days at 4 °C). The stability of DQ, DQ-D, DQ-M in rat plasma was investigated by analyzing QC samples in triplicate at two QC levels, including bench-top stability (12 h at room temperature), processed stability (24 h at autosampler, 4 °C), freeze–thaw stability (3 cycles) and long-term stability (30 days at − 70 °C).

### Toxicokinetic study in rats

All protocols were approved by the Animal Care and Ethical Committee of Nanjing Medical University. Six Sprague–Dawley rats (male, 200 ± 15 g, 6–8 weeks, Oriental BioService Inc., Nanjing, China) were housed in a fixed cycle of 12 h light–dark facility with access to standard food and water. Each rat received an intragastric administration of DQ (dissolved in normal saline) at the dose of 11 mg/kg. Whole blood samples were collected from the suborbital vein into 1.5 mL Eppendorf tubes containing Na-heparin anticoagulant before dosing (0.0 h) and at 0.0833, 0.25, 0.5, 0.75, 1, 1.5, 2, 4, 6, 8, 12, 24, 36 h after dosing. Blood samples were centrifuged at 2,000 × *g* at 4 °C for 10 min. The plasma was separated and stored at  – 70 °C until use. The mean pharmacokinetic parameters were calculated by DAS 2.0 software (Chinese Pharmacological Association, China).

## Results

**Fig.2:**
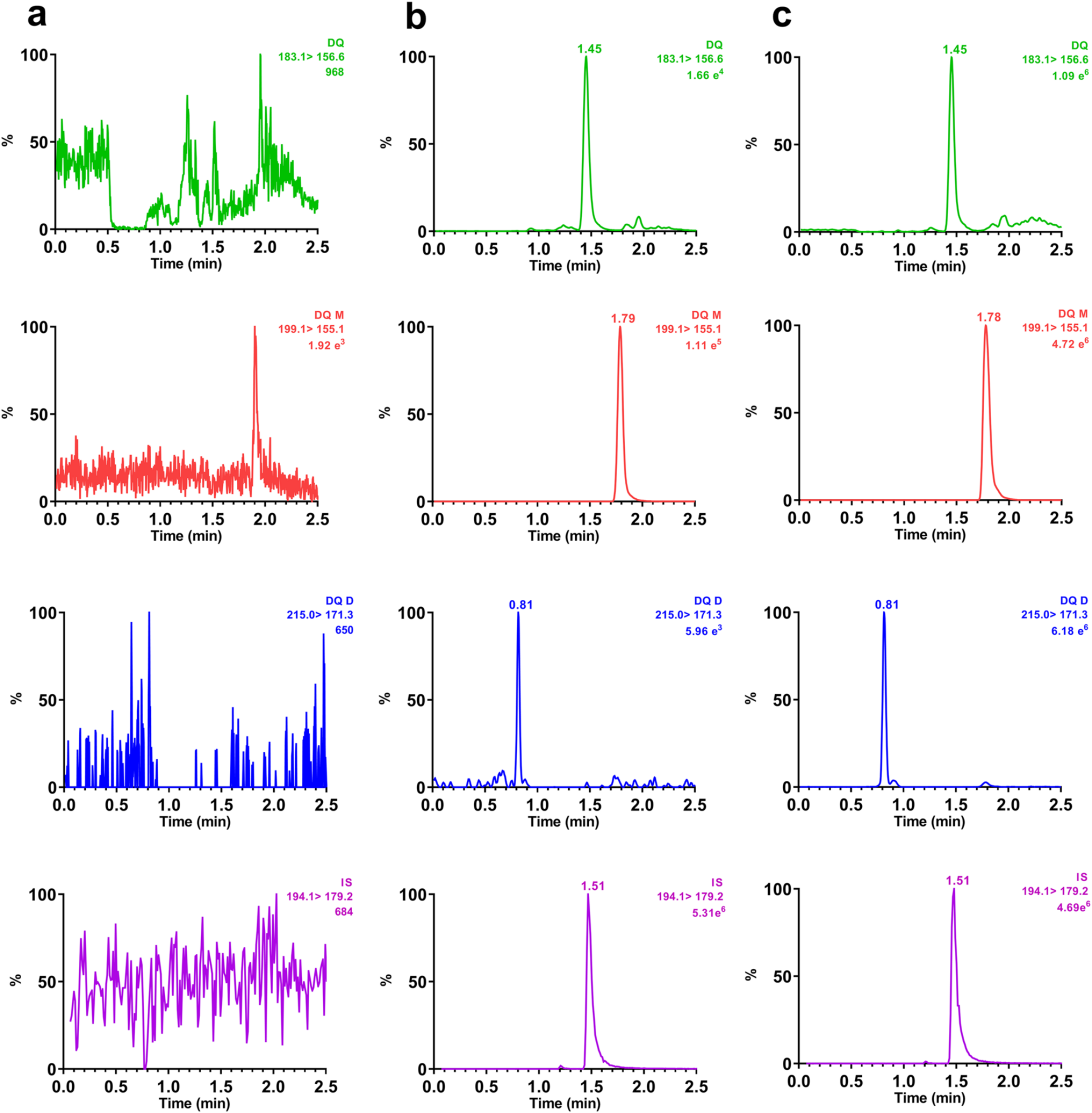
Typical multiple reaction monitoring chromatograms of DQ, DQ-M, DQ-D and IS in rat plasma. **a** blank plasma; **b** blank plasma spiked with DQ, DQ-M and DQ-D at their lower limit of quantification; **c** real plasma sample obtained from rat after intragastric administration of DQ at the dose of 11 mg/kg

The selectivity of the UPLC–MS/MS method was investigated by analyzing blank rat plasma from six different sources to ensure that there were no interfering peaks at the retention times (RTs) of all analytes. No significant interfering signals were found at the retention times of all analytes (Fig. 2). Carryover was assessed by three consecutive injections of an extracted blank sample after the injection of an extracted the upper limit of quantification sample. No peak of DQ, DQ-D, DQ-M or IS from the blank sample was observed, indicating no carryover from residues in the auto sampler.

The calibration curves were found to be linear over the specified concentration range of 3.0–3000 ng/mL for DQ, 0.3–300 ng/mL for DQ-M and 0.9–900 ng/mL for DQ-D, respectively (Table 2). The LLOQs were ranged from 0.3 to 3.0 ng/mL for DQ, DQ-D and DQ-M, which were proved to be sufficient for the toxicokinetic study in rats. The accuracy value of DQ, DQ-D and DQ-M was between −12.1 and 13.8% with the highest precision at 13.5% at three QC levels (Table S1).

**Table 2:** Regression equations, linear ranges, lower limit of quantification (LLOQ) and limit of detection (LOD) for the determination of DQ, DQ-M and DQ-D in rat plasma

Compound	Regression equation	*r*	Linear range (ng/mL)	LLOQ			LOD (ng/mL)
				Concentration (ng/mL)	RSD (%)	RE (%)	
DQ	*y* = 0.0174*x* + 0.3575	0.9971	3.00–3000	3.00	18.8	9.9	0.9
DQ-M	*y* = 0.3467*x* + 0.1277	0.9928	0.30–300	0.30	0.17	1.1	0.1
DQ-D	*y* = 0.0031*x* + 0.0003	0.9945	0.90–900	0.90	16.0	− 9.8	0.6

*RSD* relative standard deviation, *RE* relative error

The value of matrix effects ranged from 86.7 to 91.0% for DQ, 83.7 to 98.0% for DQ-M, 90.7 to 96.9% for DQ-D and 90.0% for IS, respectively. The recovery values of DQ, DQ-D and DQ-M in rat plasma were between 83.3 and 97.8% at three QC levels, and that of IS was 93.7%. The results of recovery and matrix effect are listed in Table S1.

DQ, DQ-D and DQ-M were found to be stable in stock solutions for at least 14 days at 4 °C. The deviations of the peak areas of the analytes from those of freshly prepared solutions were within ± 8.9%. In addition, DQ, DQ-D and DQ-M were stable in rat plasma for 12 h at room temperature (25 °C), for 24 h in the auto sampler at 4 °C (post preparative), after three freeze–thaw cycles and for 30 days at  – 70 °C. All stability experiment results are summarized in Table S2.

**Fig.3:**
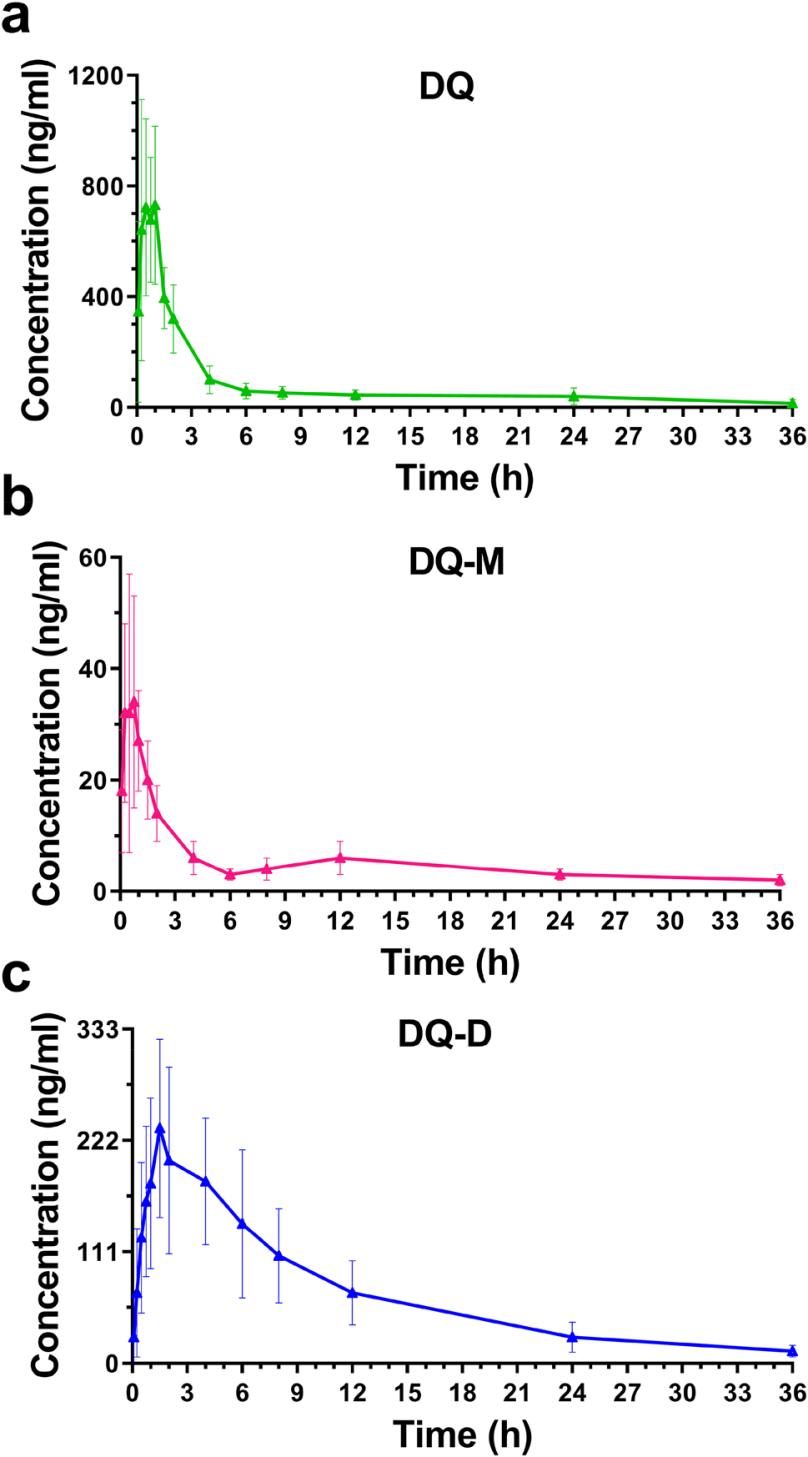
Plasma concentration–time profiles of DQ (**a**), DQ-M (**b**) and DQ-D (**c**) in rats after intragastric administration of DQ at the dose of 11 mg/kg (*n* = *6*)

This validated method was applied to the DQ, DQ-D and DQ-M toxicokinetic studies in rats after intragastric administration of DQ. The plasma concentration of DQ, DQ-D and DQ-M versus time profiles are presented in Fig. 3. The mean pharmacokinetic parameters calculated by the DAS 2.0 software are shown in Table 3. The maximum concentration (*C*_max_) was 977 ± 301 ng/mL, half-life (*t*_1/2_) was 13.1 ± 5.3 h, time to maximum concentration (*T*_max_) was 0.6 ± 0.3 h, mean residence time (MRT_0–t_) was 7.7 ± 2.4 h and area under the plasma concentration–time curve (AUC_0–t_) was 2770 ± 972 h·ng/mL for DQ, *C*_max_ was 47.1 ± 22.7 ng/mL, *t*_1/2_ was 25.1 ± 20.2 h, *T*_max_ was 0.5 ± 0.2 h, MRT_0-t_ was 10.9 ± 2.4 h and AUC_0-t_ was 180 ± 50.6 h·ng/mL for DQ-M, and *C*_max_ was 246 ± 81.8 ng/mL, *t*_1/2_ was 8.2 ± 1.5 h, *T*_max_ was 1.6 ± 0.2 h, MRT_0–t_ was 9.4 ± 1.2 h and AUC_0–t_ was 2430 ± 1040 h·ng/mL for DQ-D, respectively.

**Table 3:** The main toxicokinetic parameters of DQ, DQ-M and DQ-D in rats after intragastric administration of DQ at dose of 11 mg/kg (*n* = 6)

Toxicokinetic parameters	DQ	DQ-M	DQ-D
*C*_max_ (ng/mL)	977 ± 301	47.1 ± 22.7	246 ± 81.8
*T*_max_ (h)	0.6 ± 0.3	0.5 ± 0.2	1.6 ± 0.2
*t*_1/2_ (h)	13.1 ± 5.3	25.1 ± 20.2	8.2 ± 1.5
MRT_0–t_ (h)	7.7 ± 2.4	10.9 ± 2.4	9.4 ± 1.2
MRT_0–∞_ (h)	12.3 ± 6.6	32.6 ± 27.2	11.9 ± 2.1
AUC_0–t_ (h**·**ng/mL)	2770 ± 972	180 ± 50.6	2430 ± 1040
AUC_0–∞_ (h**·**ng/mL)	3050 ± 1180	257 ± 119	2560 ± 1090

## Discussion

DQ is a commonly used herbicide that kills both weeds and grasses because of its rapid action and low production cost. A few cases of human poisoning were reported worldwide mainly due to intentional ingestion of the liquid formulations [1]. It is well known that the kidney is the main injury organ in the DQ poisoning. Central pontine myelinolysis has been found recently in a lethal DQ poisoning case, which indicated that the potential for pontine damage should be aware of in patients with DQ poisoning [2].

The methods for determination of DQ have been developed for application in non-biological samples, such as cowpea, fruit and drinking water [4–6], and in biological samples, such as whole blood, serum, postmortem blood and urine [7–9]. All of these methods are limited to the detection of DQ and cannot simultaneously detect its metabolites. To the best of our knowledge, there are only two published studies which determined DQ and its two metabolites in biological materials by high-performance liquid chromatography (HPLC) [3, 12]. Both methods were developed using HPLC technology, which was less sensitive than LC–MS/MS.

As far as we know, the toxicokinetics of DQ-M and DQ-D have not been reported yet. To conduct toxicokinetic studies on DQ, DQ-M and DQ-D, a simultaneous analysis method of these analytes was first developed by UPLC–MS/MS. The ion intensities of DQ, DQ-M and DQ-D are all higher in ESI-positive mode than negative mode. DQ, DQ-M and DQ-D show the main singly charged cation *m/z* at 183.1, 199.1 and 215.0 (Fig. S1), respectively. Finally, the quantification *m/z* transitions of 183.1 → 156.6, 199.1 → 155.1 and 215.0 → 171.3 and qualification *m/z* transitions of 183.1 → 129.5, 199.1 → 78.3 and 215.0 → 153.3 were chosen for monitoring DQ, DQ-M and DQ-D, respectively (Table 1).

The IS should have similar behavior in terms of retention time, ionization effect, and mass spectrometer response when compared to the analytes. Generally, isotope label IS is the most ideal candidate for biological sample analysis by LC−MS/MS. However, Suzuki et al. [13] pointed out that there was a risk that DQ was produced from DQ-d4 by undergoing a deuterium–hydrogen exchange reaction and it could lead to misdetection of DQ. Therefore, in this study, paraquat-d8, a stable isotope-labeled internal standard, was selected as a desirable IS due to its similar chromatographic behavior and mass response with the analytes.

The toxicokinetic studies of DQ, DQ-M and DQ-D were performed after intragastric administration of DQ in rats. The plasma concentration–time profile showed that DQ could be rapidly absorbed after intragastric administration with the *T*_max_ of 0.6 h. The *T*_max_ of DQ-M (0.5 h) was closed with DQ, which meant that DQ-M could be produced quickly by metabolism of DQ in rats. The *T*_max_ of DQ-D (1.6 h) was longer than those of both DQ and DQ-M, which indicated that DQ-D was gradually generated by DQ or DQ-M. The *C*_max_ ratios were 0.05 for DQ-M/DQ and 0.25 for DQ-D/DQ, respectively. The maximum concentration of DQ-D in rats was 5.2 times higher than that of DQ-M, which meant that DQ-D was the primary metabolite of DQ. The *t*_1/2_ ranged from 8.2 to 25.1 h for DQ, DQ-M and DQ-D. The biodistributions of DQ-M and DQ-D were not carried out in this study which  will be studied in our future research.

## Conclusions

In this study, we developed and fully validated a rapid and reliable method for the determination of DQ and its two primary metabolites in rat plasma by UPLC−MS/MS. This method was successfully applied to the toxicokinetic studies of DQ, DQ-M and DQ-D in rats. For the first time, the toxicokinetics of DQ and its two primary metabolites was simultaneously evaluated in rats after intragastric administration of DQ at the dose of 11 mg/kg. DQ-D is the main metabolite of DQ in rats, and the maximum concentration is 5.2 times higher than that of DQ-M.

## Supplementary Information

Below is the link to the electronic supplementary material.

Supplementary file1 (DOCX 388 KB)

## References

[1] Magalhães N, Carvalho F, Dinis-Oliveira RJ (2018). Human and experimental toxicology of diquat poisoning: toxicokinetics, mechanisms of toxicity, clinical features, and treatment. Hum Exp Toxicol.

[2] Xing J, Chu Z, Han D, Jiang X, Zang X, Liu Y, Gao S, Sun L (2020). Lethal diquat poisoning manifesting as central pontine myelinolysis and acute kidney injury: A case report and literature review. J Int Med Res.

[3] Fuke C, Ameno K, Ameno S, Kinoshita H, Ijiri I (1996). Detection of two metabolites of diquat in urine and serum of poisoned patients after ingestion of a combined herbicide of paraquat and diquat. Arch Toxicol.

[4] Pizzutti IR, Vela GME, de Kok A, Scholten JM, Dias JV, Cardoso CD, Concenço G, Vivian R (2016). Determination of paraquat and diquat: LC–MS method optimization and validation. Food Chem.

[5] Oulkar D, Shinde R, Khan Z, Banerjee K (2019). High throughput residue analysis of paraquat and diquat involving hydrophilic interaction liquid chromatographic separation and mass spectrometric determination. Food Addit Contam Part A Chem Anal Control Expo Risk Assess.

[6] Hao C, Zhao X, Morse D, Yang P, Taguchi V, Morra F (2013). Optimized liquid chromatography tandem mass spectrometry approach for the determination of diquat and paraquat herbicides. J Chromatogr A.

[7] Ariffin MM, Anderson RA (2006). LC/MS/MS analysis of quaternary ammonium drugs and herbicides in whole blood. J Chromatogr B.

[8] Wang KC, Chen SM, Hsu JF, Cheng SG, Lee CK (2008). Simultaneous detection and quantitation of highly water-soluble herbicides in serum using ion-pair liquid chromatography-tandem mass spectrometry. J Chromatogr B.

[9] Tsao YC, Lai YC, Liu HC, Liu RH, Lin DL (2016). Simultaneous determination and quantitation of paraquat, diquat, glufosinate and glyphosate in postmortem blood and urine by LC-MS-MS. J Anal Toxicol.

[10] European Medicines Agency (2015) Guideline on bioanalytical method validation, London. https://www.ema.europa.eu/en/documents/scientific–guideline/guideline–bioanalytical–method–validation_en.pdf. Accessed 03/06/2015

[11] Food and Drug Administration, USA (2018) Bioanalytical method validation guidance for industry. http://www.fda.gov/files/drugs/published/Bioanalytical–method–validation–guidance–for –industry.pdf. Accessed 24/05/2018

[12] Fuke C, Arao T, Morinaga Y, Takaesu H, Ameno K, Miyazaki T (2002). Analysis of paraquat, diquat and two diquat metabolites in biological materials by high-performance liquid chromatography. Leg Med.

[13] Suzuki Y, Kaneko T, Saito K (2018). The internal standard diquat-*d*4 causes errors in diquat analysis by LC–MS/MS. Forensic Toxicol.

