# Supplementary material for: Simultaneous determination of diquat and its two primary metabolites in rat plasma by ultraperformance liquid chromatography–tandem mass spectrometry and its application to the toxicokinetic study
Source: Forensic Toxicol. 2022 Apr 12;40(2):332–9. doi: 10.1007/s11419-022-00623-z (PMC9715450; doi:10.1007/s11419-022-00623-z)
Supplement: Supplementary file 1 — Supplementary file1 (DOCX 388 KB) [file 11419_2022_623_MOESM1_ESM.docx]

**Supplementary material Fig. S1 a** Mass spectrum (left) and product ion spectrum (right) of DQ, **b** Mass spectrum (left) and product ion spectrum (right) of DQ-M, **c** Mass spectrum (left) and product ion spectrum (right) of DQ-D, **d** Mass spectrum (left) and product ion spectrum (right) of IS


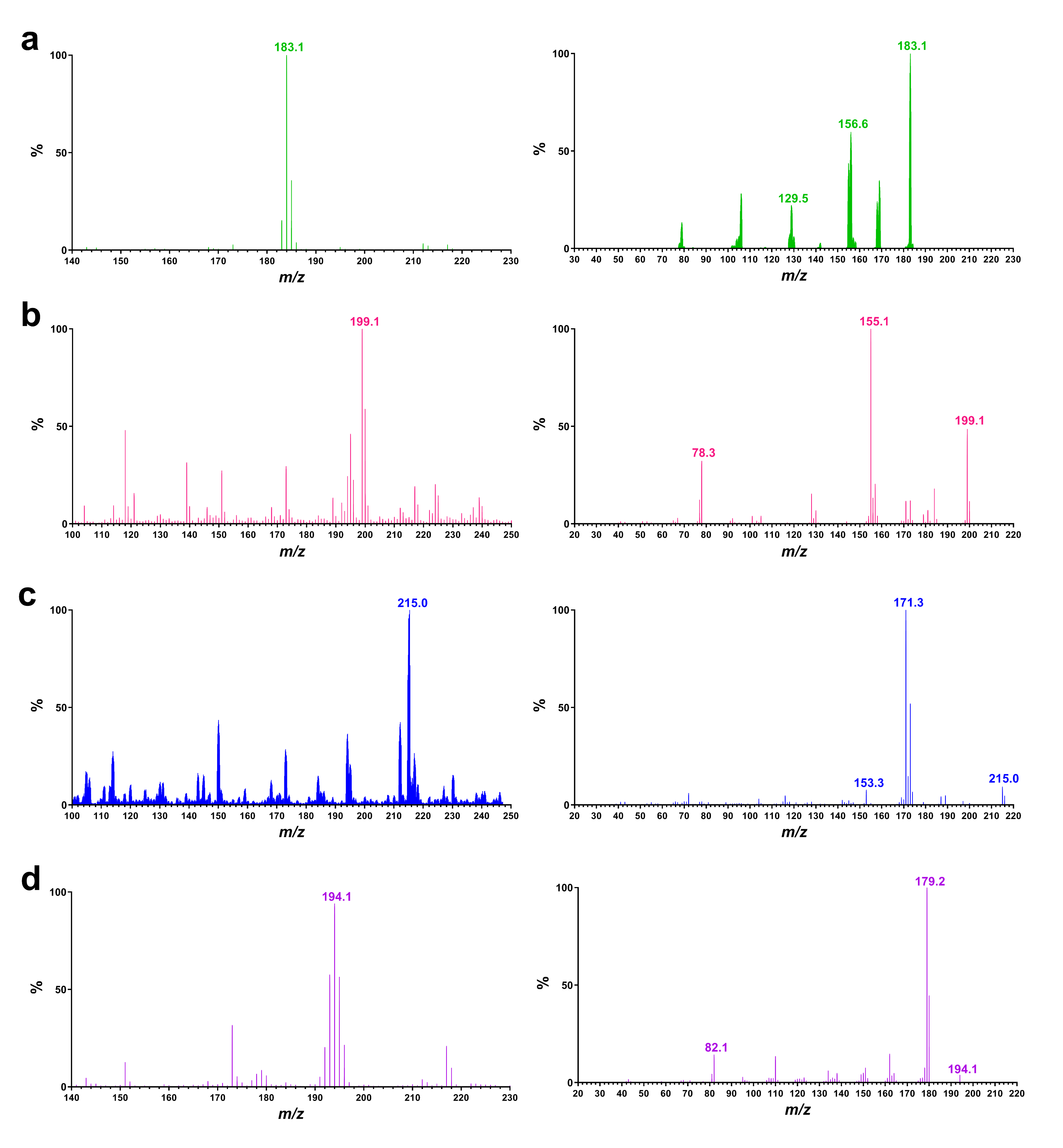


**Supplementary material Table S1** Precision, accuracy, extraction recovery and matrix effect for the determination of DQ, DQ-M and DQ-D in rat plasma

| Compound | Nominal concentration (ng/mL) | Precision | | Accuracy  (%) | Extraction recovery  (%) | Matrix effect  (%) |
| --- | --- | --- | --- | --- | --- | --- |
|  |  | Intra-day RSD (%) | Inter-day RSD (%) |  |  |  |
| DQ | 9.00 | 8.1 | 12.9 | -6.0 | 88.4 ± 3.2 | 91.0 ± 1.4 |
|  | 300 | 8.5 | 14.5 | -7.5 | 89.4 ± 1.3 | 88.4 ± 2.3 |
|  | 2700 | 7.4 | 12.3 | -5.1 | 88.3 ± 2.9 | 86.7 ± 5.9 |
| DQ-M | 0.90 | 6.9 | 11.2 | 8.2 | 94.5 ± 3.6 | 83.7 ± 6.5 |
|  | 90 | 10.7 | 7.3 | -5.3 | 94.9 ± 1.0 | 95.7 ± 1.7 |
|  | 270 | 4.6 | 13.5 | -3.5 | 95.1 ± 3.3 | 98.0 ± 6.4 |
| DQ-D | 3.00 | 5.5 | 13.1 | 13.8 | 97.8 ± 7.2 | 90.7 ± 1.6 |
|  | 300 | 9.1 | 9.9 | -1.1 | 87.2 ± 10.8 | 93.9 ± 1.2 |
|  | 810 | 10.8 | 9.6 | -12.1 | 83.3 ± 5.6 | 96.9 ± 3.6 |
| IS | 500 |  |  |  | 93.7 ± 7.0 | 90.0 ± 3.4 |

**Supplementary material Table S2** Stability results for DQ, DQ-M and DQ-D in rat plasma under different conditions

| Storage condition | DQ | |  | DQ-M | |  | DQ-D | |
| --- | --- | --- | --- | --- | --- | --- | --- | --- |
|  | Concentration (ng/mL) | RE (%) |  | Concentration (ng/mL) | RE (%) |  | Concentration (ng/mL) | RE (%) |
| Stock solution stability | 9.00 | 1.5 |  | 0.90 | -8.9 |  | 3.00 | 7.3 |
|  | 2700 | -1.0 |  | 270 | -2.8 |  | 810 | 4.7 |
| Bench-top stability | 9.00 | -3.3 |  | 0.90 | 2.6 |  | 3.00 | 13.1 |
|  | 2700 | -5.1 |  | 270 | 3.0 |  | 810 | -11.6 |
| Processed sample stability | 9.00 | -6.1 |  | 0.90 | -6.2 |  | 3.00 | -2.9 |
|  | 2700 | -2.7 |  | 270 | 3.5 |  | 810 | 2.7 |
| Freeze and thaw stability | 9.00 | -3.9 |  | 0.90 | -6.6 |  | 3.00 | 5.6 |
|  | 2700 | -3.2 |  | 270 | 0.6 |  | 810 | -6.2 |
| Long term stability | 9.00 | -10.5 |  | 0.90 | -0.2 |  | 3.00 | -14.3 |
|  | 2700 | -8.1 |  | 270 | 7.7 |  | 810 | 11.7 |
